# Sequence and directivity in cardiac muscle injury of COVID-19 patients: an observational study

**DOI:** 10.3389/fcvm.2023.1260971

**Published:** 2023-10-16

**Authors:** Yixuan Wang, Jianxiong Chen, Lin Jin, Lingheng Wu, Mengjiao Zhang, Jiali Sun, Cuiqin Shen, Lianfang Du, Bei Wang, Zhaojun Li

**Affiliations:** ^1^ Department of Medical Ultrasound, Shandong Medicine and Health Key Laboratory of Abdominal Medical Imaging, The First Affiliated Hospital of Shandong First Medical University & Shandong Provincial Qianfoshan Hospital, Jinan, China; ^2^ Department of Ultrasound, Shanghai General Hospital, Shanghai Jiaotong University School of Medicine, Shanghai, China; ^3^ Department of Ultrasound, Shanghai General Hospital of Nanjing Medical University, Shanghai, China; ^4^ Department of Ultrasound, Guanghua Hospital Affiliated to Shanghai University of Traditional Chinese Medicine, Shanghai, China; ^5^ Department of Ultrasound, Jiading Branch of Shanghai General Hospital, Shanghai Jiaotong University School of Medicine, Shanghai, China

**Keywords:** COVID-19 infection, left ventricular global longitudinal strain, echocardiography, speckle-tracking, heart injuries

## Abstract

**Objective:**

To compare cardiac function indicators between mild and moderate to severe COVID-19 patients and to try to identify the sequence and directivity in cardiac muscle injury of COVID-19 patients.

**Methods:**

From December 2022 to January 2023, all patients with laboratory-confirmed SARS-CoV-2 infection in Shanghai General Hospital Jiading Branch were enrolled. The clinical classification was stratified into mild, moderate, or severe groups. We collected the clinical and laboratory information, transthoracic echocardiographic and speckle-tracking echocardiographic parameters of patients and compared the differences among different groups.

**Results:**

The values of echocardiographic parameters in mild group were lower than that in moderate or severe group (*P* < 0.05) except LVEF. The values of LVEF of mild and moderate group were higher than severe group (*P* < 0.05). There were no significant differences between moderate and severe group. Positive correlations were observed between left ventricular global longitudinal strain (LVGLS) and myoglobin (*r* = 0.72), E/e' and age (*r* = 0.79), E/e' and BNP (*r* = 0.67). The multivariate analysis shows that SpO_2_ (OR = 0.360, *P* = 0.02), LVGLS (OR = 3.196, *P* = 0.003) and E/e' (OR = 1.307, *P* = 0.036) were the independent risk factors for mild cases progressing to moderate or severe. According to the receiver operating characteristic (ROC) curves, when all the COVID-19 patients was taken as the sample size, the area under the curve (AUC) of the LVGLS was the highest (AUC = 0.861). The AUC of the LVGLS was higher than LVGCS (AUC = 0.565, *P* < 0.001).

**Conclusion:**

When mild COVID-19 progresses to moderate or severe, both systolic and diastolic functions of the heart are impaired. LVGLS was the independent risk factor for mild cases progressing to moderate or severe cases. Longitudinal changes may manifest earlier than circumferential changes as myocardial disease progresses in COVID-19.

## 1. Introduction

Since its emergence in December 2019, COVID-19 has rapidly become a global health concern. While primarily recognized for its respiratory impact, it is increasingly evident that COVID-19 can exert a significant influence on the cardiovascular system. This impact encompasses a spectrum of cardiovascular diseases, including but not limited to myocarditis, acute coronary syndrome, arrhythmias, and heart failure (1). Some studies have proved that patients with COVID-19 related myocardial injury, which was associated with death (2, 3). Understanding the intricate relationship between COVID-19 and these cardiovascular complications is of paramount importance in the field of cardiovascular medicine. However, few studies have been conducted on cardiac function. Cardiac function includes systolic and diastolic functions. Echocardiography is the most commonly used diagnostic tool for evaluating cardiac function. Ultrasonography, including conventional and bedside ultrasound, plays an important role in diagnosing diseases in COVID-19 patients (4, 5). In addition to conventional ultrasound, two-dimensional speckle tracking technology has been widely applied in recent years to detect early abnormalities in ventricular function and accurately assess myocardial function (6, 7). Previous studies have reported a decrease in left ventricular global longitudinal strain (LVGLS) in COVID-19 patients (8). However, there was almost none study has compared cardiac function in patients with different degrees of disease severities. Therefore, the purpose of this study was to compare cardiac function indicators in patients with mild and moderate-to-severe COVID-19 and to identify alterations in cardiac function during the progression of COVID-19.

## 2. Materials and methods

### 2.1. Study population

This study was an observational study. All patients with laboratory-confirmed SARS-CoV-2 infection at Shanghai General Hospital Jiading Branch from December 2022 to January 2023 were included in this study. The inclusion criteria were: (1) age >18 years and (2) COVID-19 diagnosis by RT-PCR swab test and comprehensive analysis of epidemiological history and clinical presentation. Patients who were <18-years-old and with end-stage renal failure, active malignancy, previous or current autoimmune diseases, coronary atherosclerotic heart disease, or congenital heart disease were excluded. This study got the permission of the Ethics Committee of Shanghai General Hospital (2019KY009-4) and complied with the Declaration of Helsinki. Every patient signed informed consent.

### 2.2. Definition of mild, moderate, and severe COVID-19 infection

Clinical classification followed the Diagnosis and Treatment Protocol of Novel Coronavirus (Version 10) by the National Health Commission of the People's Republic of China (9), which stratifies patients according to disease severity as mild, moderate, or severe.

Mild: The main symptoms are upper respiratory tract infections, such as dry throat, sore throat, cough, and fever.

Moderate: Persistent high fever for more than 3 days (and/or) coughing, shortness of breath, etc., but respiratory rate (RR) is <30 breaths/min and oxygen saturation is >93% when air is inhaled at rest. The imaging features of COVID-19 pneumonia were observed.

Severe: Adults satisfying any of the following factors that cannot find other reasons other than COVID-19:

1. Shortness of breath, RR is ≥30 breaths/min;
2. Oxygen saturation is ≤93% when inhaling air at rest;
3. Arterial oxygen partial pressure (PaO_2_)/oxygen uptake concentration (FiO_2_) is ≤300 mmHg (1 mmHg = 0.133 kPa);
4. The clinical symptoms gradually worsen, and pulmonary medical imaging results shows significant progression within 24–48 h, with a rate of >50%.

### 2.3. Data collection

#### 2.3.1. Clinical and laboratory information

The following information was collected: age, sex, height, body mass index (BMI), heart rate, arterial systolic and diastolic blood pressure, and saturation of peripheral oxygen (SpO_2_). Medical histories of diabetes, hypertension, and use of antihypertensive medications were recorded. The BMI was calculated as weight/height^2^ (kg/m^2^). Laboratory parameters included C-reactive protein (CRP), B-type Natriuretic Peptide (BNP), cardiac troponin I (cTnI), creatine kinase isoenzyme (CKMB), and myoglobin.

#### 2.3.2. Transthoracic echocardiography

A Philips EPIQ7 echocardiograph equipped with an S5-1 probe (frequency 1–5 MHz) and frame rate ≥60 beats/min was used. When performing echocardiography, patients should remain in the left lateral position and maintain calm breathing. A synchronized electrocardiogram was connected to an ultrasound machine. The left ventricular end diastolic diameter (LVDd), left ventricular end systolic diameter (LVDs), interventricular septum diameter (IVSd) and left ventricular posterior wall diameter (LVPWd) were measured in each patient, representing the end-diastolic/systolic anteroposterior diameter of left ventricular, left ventricular end-diastolic septal thickness, and left ventricular end-diastolic posterior wall thickness, respectively. The ratio of E/A and E/e' were used to estimate LV diastolic function, where E and A are early and late transmitral flow velocities, and e' is early diastolic septal tissue velocity. Left ventricular ejection fraction (LVEF) was measured using the modified Simpson biplane method.

#### 2.3.3. Speckle-tracking echocardiographic analysis

**Figure 1:**
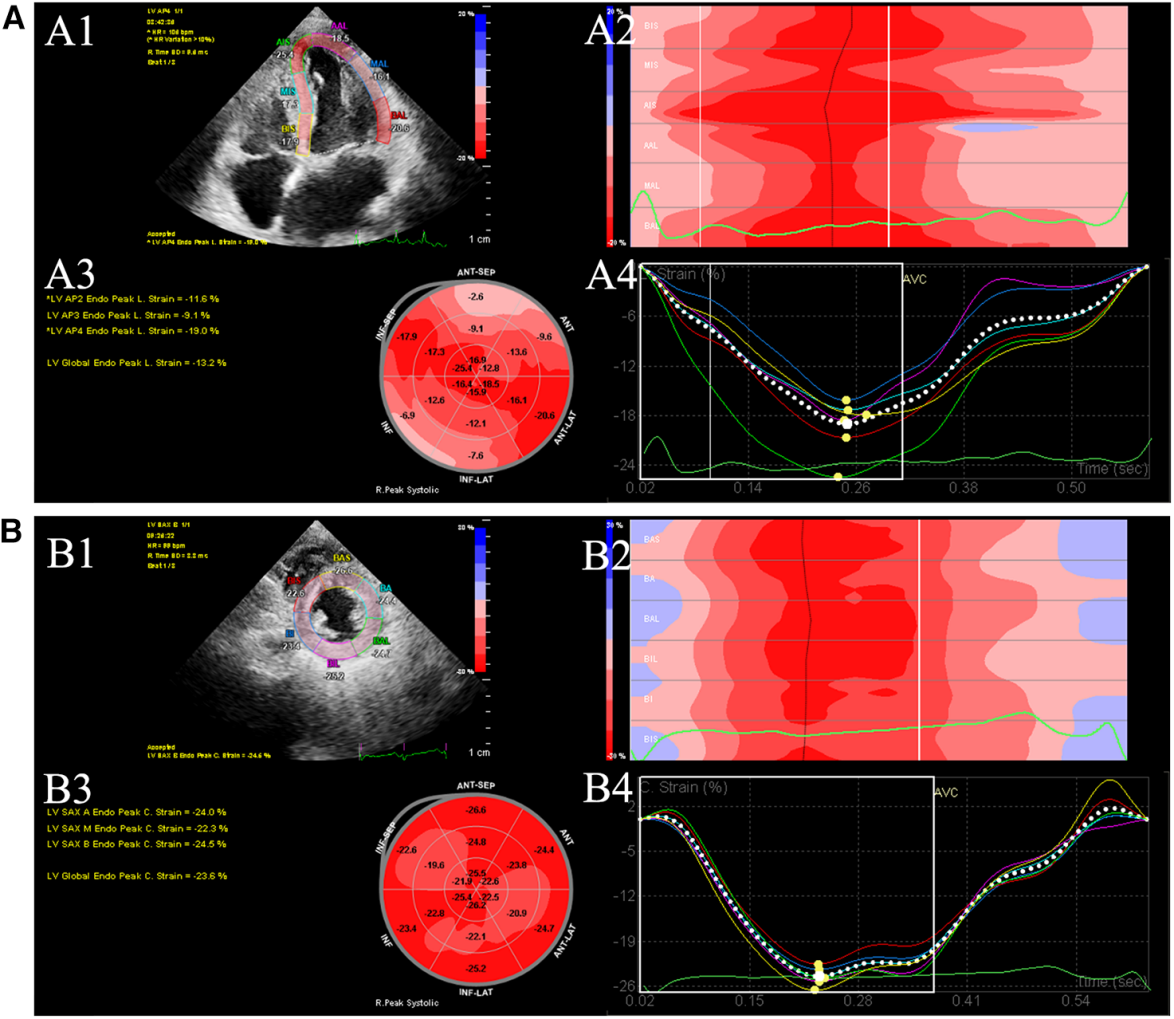
Offline analysis of GLS (**A**) and GCS (**B**) of left ventricular (LV). (**A1**) LV endocardial tracing in the LV apical 4-chamber. (**A2**) Longitudinal peak strain of 6 segments. (**A3**) 18-segment bull's eye diagram of LVGLS. (**A4**) GLS strain curves were generated. (**B1**) LV endocardial tracing in the LV short-axis views. (**B2**) Circumferential peak strain of 6 segments. (**B3**) 18-segment bull's eye diagram of GCS. (**B4**) LVGCS strain curves were generated. GCS global circumferential strain. GLS global longitudinal strain. LV left ventricular.

Two-dimensional videos of three consecutive cardiac cycles were collected at the end of breathing for post-offline analysis. In addition, the images of apical 4-chamber, 2-chamber, 3-chamber, and short-axis views were saved at the level of the parasternal papillary muscle. As shown in Figure 1, all the images were processed with Qlab13 (2D AutoStrain, Philips Healthcare). Left ventricular global longitudinal strain (LVGLS) and left ventricular global circumferential strain (LVGCS) were measured and stored. The measurements and calculated formulas for the parameters were conducted according to the recommendations in 2015 (10). In this study, we defined LVGLS imaging and value below −18% was consider as abnormal (11).

#### 2.3.4. Reproducibility test

Two physicians independently assessed LVGLS and LVGCS values for a cohort of 20 randomly selected participants to evaluate the reproducibility of the measurements. The assessment of inter-observer agreements between the two physicians was conducted using Bland–Altman plots.

### 2.4. Statistical analysis

Enumeration data were expressed as numbers and percentages, measurement data were presented as mean and standard deviation, or expressed as median (interquartile range) for quantitative variables. Data analysis was performed using SPSS 23.0 (IBM, Armonk, NY, USA). One-way ANOVA and Bonferroni correction were used for intergroup and intragroup comparison of continuous data, respectively. Non-normally distributed data were analyzed by the Wilcoxon signed-rank test, and categorical variables between the two groups were compared by chi-square tests. A linear correlation analysis and Bland–Altman plots were adopted for repeatability evaluation. The correlation between different parameters was analyzed by Spearman's method.

To analyze the risk factors associated with moderate/severe COVID-19, a backward stepwise regression method was employed. The variables included in the analysis were clinical data such as age, sex, HR, SBP, SpO_2_, cTnI, Myoglobin, E/A ratio, E/e' ratio, LVGLS, and LVGCS.

The receiver operating characteristic (ROC) curve best reflected the sensitivity and specificity of the echocardiographic parameters in diagnosing moderate and severe COVID-19 at the upper leftmost value, and the area under the ROC curve (AUC) of each parameter was obtained. The value of *P* < 0.05 indicated that the difference was statistically significant.

## 3. Results

### 3.1. Clinical characteristics

Ultimately, a total of 219 COVID-19 patients were comprehensively enrolled in the study of which 101 (46%) were males and 118 (54%) were females. The demographic details of the COVID-19 patients are shown in Table 1. Of the 219 included patients, 130 (59%) had mild disease, 41 (19%) had moderate disease and 48 (22%) had severe disease. Significant differences between the groups were found in sex, age, DBP, HR, SpO2, and medical history and use of medications for diabetes and hypertension. Height, BMI, and SBP did not differ significantly between groups.

**Table 1:** Baseline characteristics of participants with COVID-19 (*n* = 219).

Variables	Mild (*n* = 130)	Moderate (*n* = 41)	Severe (*n* = 48)	F/H/χ^2^	*P* value
Sex (male %)	46 (35.4)	24 (58.5)^a^	31 (64.6)^a^	15.158	0.001
Age (years)	44.44 ± 15.65	77.15 ± 9.79^a^	83.31 ± 9.45^a^	189.195	<0.001
Height (cm)	164.72 ± 7.78	164.93 ± 7.64	166.06 ± 7.61	0.534	0.587
BMI (kg/m^2^)	23.54 ± 3.4	23.13 ± 4.35	23.1 ± 3.69	0.353	0.703
SBP (mmHg)	124.45 ± 16.3	126.7 ± 20.94	127.95 ± 19.77	0.693	0.501
DBP (mmHg)	79.76 ± 11.6	75.83 ± 14.2	73.77 ± 14.87^a^	4.027	0.019
HR (betas/min)	78.95 ± 12.29	80.6 ± 12.26	88 ± 17.36^a,b^	7.229	0.001
SpO_2_ (%)	98 (97, 99)	96 (94.25, 97.75)^a^	93 (91, 95)^a,b^	74.457	<0.001
Diabetes (%)	123 (94.6)	33 (80.5)^a^	41 (85.4)^a,b^	8.285	0.016
Anti-hypertension drugs (%)	26 (20.6)	28 (68.3)^a^	31 (64.6)^a^	45.611	<0.001
LVDd (mm)	44.87 ± 3.7	45.62 ± 4.14	45.68 ± 5.18	0.938	0.393
LVDs (mm)	27.31 ± 2.77	30.03 ± 3.4^a^	30.37 ± 3.98^a^	22.121	<0.001
IVSd (mm)	9.37 ± 0.96	9.93 ± 1.26^a^	10.46 ± 1.42^a^	17.287	<0.001
LVPWd (mm)	8.95 ± 0.89	9.68 ± 1.14^a^	9.81 ± 0.92^a^	19.086	<0.001
LVEF	70.18 ± 4.55	63.04 ± 5.22^a^	61.62 ± 6.64^a^	61.794	<0.001
E (cm/s)	78.61 ± 18.12	65.55 ± 19.75^a^	70.37 ± 29.4	6.848	0.001
A (cm/s)	66.94 ± 21.01	93.04 ± 26.08^a^	98.04 ± 21.12^a^	44.035	<0.001
E/A	1.34 ± 0.7	0.75 ± 0.29^a^	0.74 ± 0.32^a^	27.263	<0.001
E/e’	6.3 ± 2.12	10.21 ± 4.4^a^	11.38 ± 5.44^a^	42.826	<0.001
LVGLS	−22.27 ± 2.63	−18.41 ± 2.85^a^	−18.81 ± 2.96^a^	40.477	<0.001
LVGCS	−27.45 ± 3.27	−26.91 ± 3.88	−24.95 ± 5.63^a^	5.330	0.006
CRP (mg/L)	0 (0, 0)	4.97 (2.17, 10.01)^a^	17.56 (5.32, 55.61)^a,b^	92.774	<0.001
BNP (pg/ml)	19.4 (9.05, 35.7)	48.6 (29.65, 142.25)^a^	80.2 (54, 177.9)^a,b^	42.290	<0.001
cTnI (g/L)	0 (0, 0)	0 (0, 0.02)	0.02 (0, 0.05)^a,b^	19.959	<0.001
CKMB (ng/ml)	0.8 (0.43, 1.08)	0.86 (0.21, 1.99)	1.35 (0.36, 3.03)^a,b^	8.895	0.012
Myoglobin (µg/L)	22.03 (17.06, 28.42)	50.6 (32.47, 86.49)^a^	66.78 (37.86, 145.53)^a,b^	76.995	<0.001

Data are shown as mean ± SD or median (interquartile range), number and percent (%) of event outcomes. 1 mmHg = 0.133 kPa.

^a^ *P* < 0.05, compared with the mild group.

^b^ *P* < 0.05, compared with the moderate group.

### 3.2. Echocardiographic parameters

Table 1 shows all the echocardiographic parameters in the three groups. There was no significant difference in LVDd between the three groups. Besides, the values of LVDs, IVSd, LVPWd, E, A, E/A ratio, E/e' ratio, and GLS in mild group were lower than those in the moderate and severe groups (*P* < 0.05). The values of LVEF of mild and moderate group were higher than severe group (*P* < 0.05). There were no significant differences between the moderate and severe groups. Moderate and severe COVID-19 patients presented with impaired LVGLS compared to mild [mild: −22.27% (2.63) vs. moderate: −18.41% (2.85) vs. severe: −18.81% (2.96), *P *< 0.001]. Out of 219 patients, 31 (14%) had reduced LVGLS values of ≤18%.

### 3.3. Reproducibility analysis

The reproducibility of the LVGLS and LVGCS was assessed in 20 randomly selected participants (patients or controls), and these values were measured independently by two physicians. There was favorable agreement between the quantitative variables measured by them for LVGLS and LVGCS scores. The mean (±SD) difference was 0.22 (±0.80) for repeated measurements of LVGLS by the two observers. The mean (±SD) difference was 1.34 (±2.43) for repeated measurements of LVGCS by them (Supplementary Material Figure S1).

### 3.4. Laboratory examination

CRP, BNP, cTnI, CK-MB, and myoglobin levels differed significantly among the different groups (Table 1). The severe group had significantly higher levels of CRP, BNP, cTnI, CK-MB, and myoglobin than the other groups. The moderate group also had significantly higher levels of these biomarkers than the mild group except cTnI and CKMB.

### 3.5. Correlations between echocardiographic and clinical characteristics

**Figure 2:**
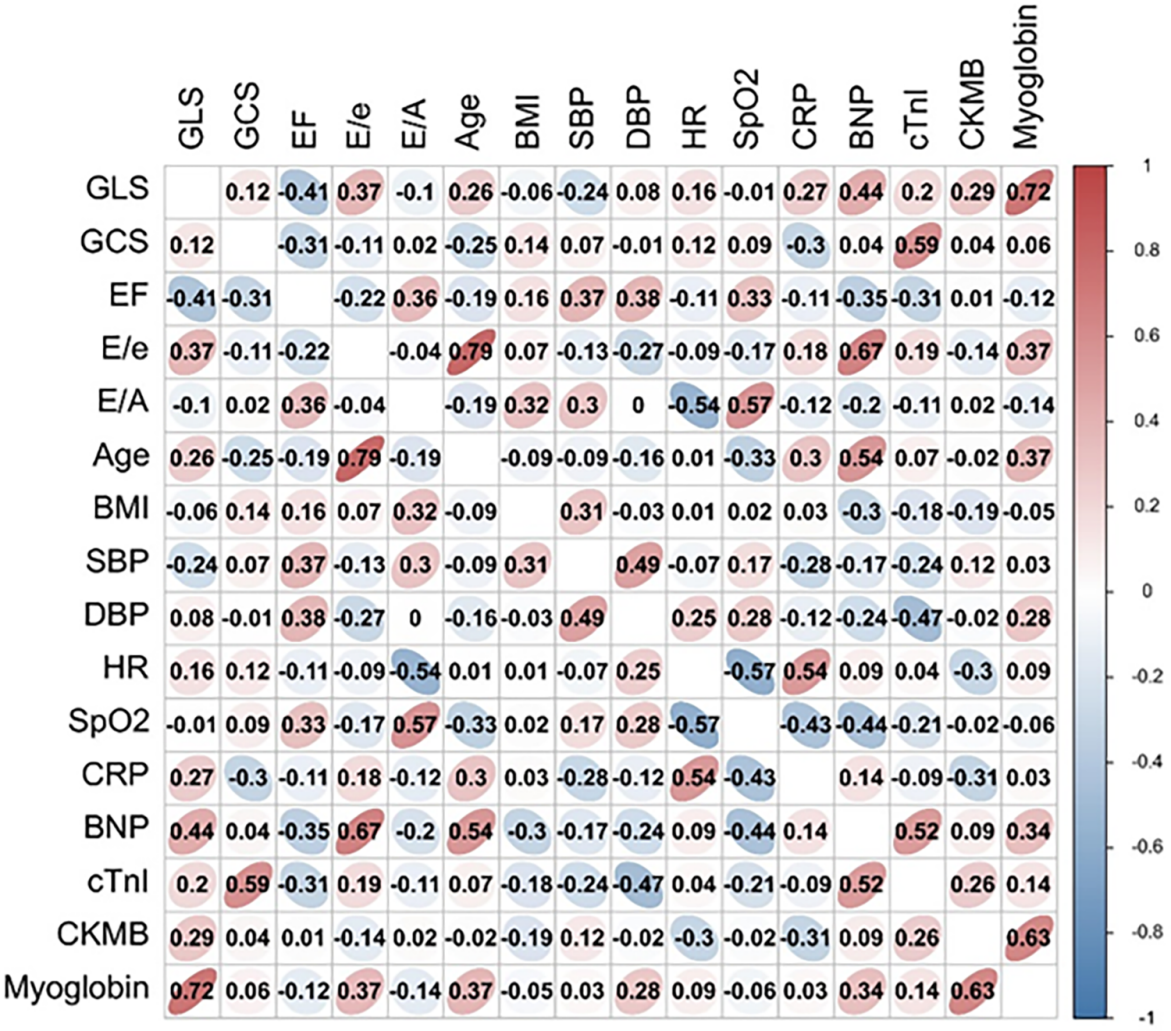
Associations between echocardiographic parameters and clinical characteristics. Positive correlations were observed between left ventricular global longitudinal strain (LVGLS) and myoglobin (*r* = 0.72), E/e’ and age (*r* = 0.79), E/e’ and BNP (*r* = 0.67). Negative correlation was found between E/A and heart rate (*r* = −0.54).

LVGLS and E/A were correlated with myoglobin and heart rate, respectively, with r values of 0.72 and −0.54 (all *P* < 0.05). E/e' was correlated with age and BNP (*r* = 0.79, 0.67; all *P* < 0.05) (Figure 2).

### 3.6. Multivariate analysis of risk factors for mild cases developing into moderate or severe cases

Because clinical characteristics, such as age and sex, were significantly different among the three groups, we conducted a multivariate logistic regression analysis of the risk factors for mild cases developing into moderate or severe cases. SpO_2_, LVGLS, and E/e' were independently associated with the progression from mild to moderate/severe cases, with ORs of 0.360, 3.196, and 1.307, respectively (*P* = 0.02, 0.003, and 0.036; Table 2).

**Table 2:** Multivariate logistic regression model analysis of risk factors for mild and moderate/severe COVID-19 (*n* = 219).

Item	*β*	Wald	*P*	OR	95% CI
SpO_2_ (%)	−1.022	9.982	0.002	0.360	0.191–0.678
SBP (mmHg)	−0.044	2.209	0.137	0.957	0.902–1.014
LVGLS	1.162	9.081	0.003	3.196	1.501–6.806
E/e’	0.268	4.399	0.036	1.307	1.018–1.68

OR, odds ratio; CI, confidence interval.

### 3.7. ROC curve of echocardiographic parameters for predicting moderate/severe diseases

**Figure 3:**
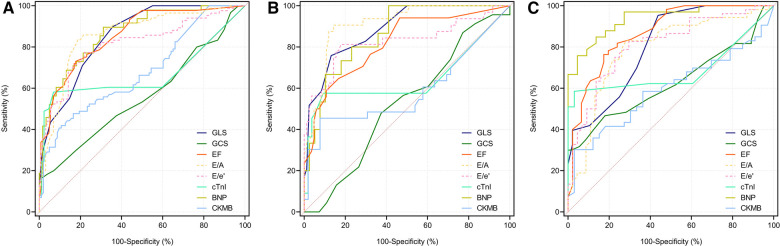
The ROC curve was used to evaluate the ability of echocardiographic parameters in diagnosing moderate and severe COVID-19 patients. Longitudinal strain parameters show negative values as ventricular myocardium shortens along the longitudinal axis during systolic activity. For easier comparison, values of EF, E/e’ and E/A were modified by changing the algebraic sign. In All subjects (**A**), female group (**B**) and male group (**C**).

ROC curves were depicted to evaluate the capacity of the echocardiographic parameters to diagnose moderate/severe COVID-19 cases (Figure 3). According to the ROC curves, when all the COVID-19 patients in our study were considered as the sample size, the AUC of the LVGLS was the highest (AUC = 0.861). The AUC of the LVGLS was higher than that of the LVGCS (Table 3; AUC = 0.565, *P* < 0.001). This indicates that the LVGLS is an excellent diagnostic tool for identifying moderate/severe diseases.

**Table 3:** The ROC curve was used to evaluate the ability of echocardiographic parameters in diagnosing moderate and severe COVID-19 patients.

	AUC	95% CI	*P* value	*Z* value	95% CI	*P* value
Overall						
LVGLS	0.861	0.802–0.908	<0.001	6.3	0.204–0.389	<0.0001^a^
LVGCS	0.565	0.489–0.638	0.947	/	/	/
LVEF	0.84	0.778–0.890	<0.001	5.123	0.170–0.381	<0.0001^b^
E/A	0.834	0.772–0.885	<0.001	4.610	0.155–0.384	<0.0001^c^
E/e’	0.841	0.780–0.891	<0.001	4.643	0.160–0.393	<0.0001^d^
Female						
LVGLS	0.85	0.766–0.912	<0.001	4.730	0.187–0.452	<0.0001^a^
LVGCS	0.53	0.429–0.628	0.802	/	/	/
LVEF	0.807	0.718–0.878	<0.001	3.474	0.121–0.474	0.0005^b^
E/A	0.89	0.813–0.943	<0.001	4.771	0.212–0.508	<0.0001^c^
E/e’	0.843	0.759–0.907	<0.001	3.615	0.143–0.483	0.0003^d^
Male						
LVGLS	0.788	0.675–0.876	<0.001	3.034	0.093–0.432	0.0024^a^
LVGCS	0.525	0.403–0.645	0.588	/	/	/
LVEF	0.865	0.763–0.935	<0.001	4.671	0.197–0.482	<0.0001^b^
E/A	0.737	0.619–0.835	0.001	2.193	0.0225–0.401	0.0283^c^
E/e’	0.829	0.721–0.908	<0.001	3.404	0.129–0.479	0.0007^d^

^a^ The *P* value indicated the comparision between ROC of LVGLS and LVGCS.

^b^ The *P* value indicated the comparision between ROC of LVEF and LVGCS.

^c^ The *P* value indicated the comparision between ROC of E/A and LVGCS.

^d^ The *P* value indicated the comparision between ROC of E/e’ and LVGCS.

## 4. Discussion

In this study, we have observed that the progression of mild cases to either moderate or severe manifestations was associated with an impairment in both systolic and diastolic cardiac functions. These changes may be related to myoglobin levels, age, BNP levels, and heart rate. The AUC of the LVGLS was higher than that of the LVGCS, indicating that the systolic function in the long-axis direction was damaged drastically. SpO_2_, LVGLS, and E/e' were the independent risk factors for mild cases progressing to moderate or severe cases. In predicting moderate/severe disease, LVGLS exhibited a higher AUC compared to LVGCS, implying an earlier onset of systolic dysfunction in the long-axis direction vs. the short-axis direction.

Cardiovascular disease remains the main cause of mortality worldwide (12, 13). Few studies have assessed cardiac function in COVID-19 patients with varying severity and identified the risk factors for mild cases worsening into moderate or severe cases. Many previous studies have shown that patients with COVID-19 can experience myocardial damage, either directly due to viral infiltration, indirectly due to respiratory failure, a systemic inflammatory response, or a combination of all three conditions (14). Patients with acute COVID-19 pneumonia without prior echocardiographic abnormalities can develop echocardiographic abnormalities, most commonly left and/or right ventricular dysfunction (15, 16).

Echocardiography along with LVGLS imaging is a handy and effective tool to ascertain early left ventricular dysfunction which is often seen in myocardial injury. Some studies have shown that left ventricular systolic and diastolic functions are impaired in COVID-19 patients, including increased left ventricular internal dimension diastole, reduced LVEF, increased left atrial volume, increased peak tricuspid regurgitation velocity (Vmax), and changes in mitral E/e' ratio (16–23).

Our study found that the reduction of systolic and diastolic functions has correlation with the severity of the illness. The parameters of left ventricular structure or systolic/diastolic functions in the mild group were lower than those in the moderate or severe groups. One study showed that conventional echocardiographic parameters of the left ventricular structure or systolic/diastolic functions did not differ between the moderate and severe groups (24). The results of the present study is consistent with this finding. For E/A ratio, there was no significantly difference between mild and severe patients (25). However, in our study, E/A ratio and LVEF was lower in moderate and severe patients compared with milder ones. This may be due to the older age of moderate to severe patients compared to mild patients.

Speckle-tracking echocardiography is good at assessing myocardial deformation because it is not affected by angle (26, 27). Longitudinal and circumferential changes diverge as myocardial disease progresses, which may contribute to the ability of strain to predict outcomes. Two-dimensional speckle tracking technology can identify myocardial dysfunction and subclinical myocardial injury (28), and studies have shown the potential value of ventricular strain assessment in COVID-19 patients (29–31). Our study found that SpO_2_, E/e' and LVGLS were the independent risk factors for mild patients progressing to moderate/severe group, and LVGLS played the most important part. The classification criteria included oxygen saturation, so SpO_2_ could influence the degree of disease. In our study, E/e’ ratio was higher in moderate and severe patients compared with milder ones which was similar to previous studies (32, 33). Lupu et al. (34) found that E/e' had the added predictive value which could improve outcome prediction.

Myocardial longitudinal strain mainly reflects cardiac contractile function, while myocardial circumferential strain is more closely related to cardiac preload (35). In this study, the systolic function decreased more significantly than the diastolic function. Moreover, LVGLS proved to be a more effective predictor for diagnosing moderate/severe cases of COVID-19 than LVGCS. This superiority in predictive ability may be attributed to its reflection of the function of longitudinally oriented subendocardial muscle fibers, which are adversely affected in the early stages of myocardial injury. A similar situation was observed during COVID-19 infection, with a significant decrease in LVGLS in severe patients (36). A recent meta-analysis of critically ill patients with severe sepsis and septic shock showed that left ventricular GLS is a reliable marker of left ventricular systolic function (37). Another meta-analysis showed that lower LVGLS was independently associated with adverse outcomes of COVID-19. Subgroup analysis showed that for every 1% decrease in LVGLS, the mortality rate increased by 1.3 times (31).

A limitation of our study was that it was a single-center study and recruited a small number of patients. Second, the patients in the mild group were younger than those in the moderate/severe groups. Age differences may also affect cardiac function. However, we conducted logistic regression analysis to identify age as a risk factor for heart disease. Besides, we did not set normal control patients group. In addition, we did not observe the normalization of echocardiographic data after recovery. We intend to continue this research in the future.

## 5. Conclusion

When mild COVID-19 progresses to moderate or severe, both systolic and diastolic functions of the heart are impaired. LVGLS was the independent risk factor for mild cases progressing to moderate or severe cases. Longitudinal changes may manifest earlier than circumferential changes as myocardial disease progresses in COVID-19.

## Data Availability

The raw data supporting the conclusions of this article will be made available by the authors, without undue reservation.

## Supplementary material

Supplementary Figure 1 The repeatability evaluation adopted a linear correlation analysis and Bland-Altman plots. (**A**) The mean (±SD) difference was 0.22 (±0.80) for repeated measurements of GLS taken by two independent observers. (**B**) The linear correlation of GLS between 2 observers showed that *R*^2^ = 0.87. (**C**) The mean (±SD) difference was 1.34 (±2.43) for repeated measurements of GCS taken by two independent observers. (**D**) The linear correlation of GCS between 2 observers showed that *R*^2^ = 0.65.
