# Supplementary figures and images for: Sequence and directivity in cardiac muscle injury of COVID-19 patients: an observational study
Source: Front Cardiovasc Med. 2023 Oct 16;10:1260971. doi: 10.3389/fcvm.2023.1260971 (PMC10613984; doi:10.3389/fcvm.2023.1260971)

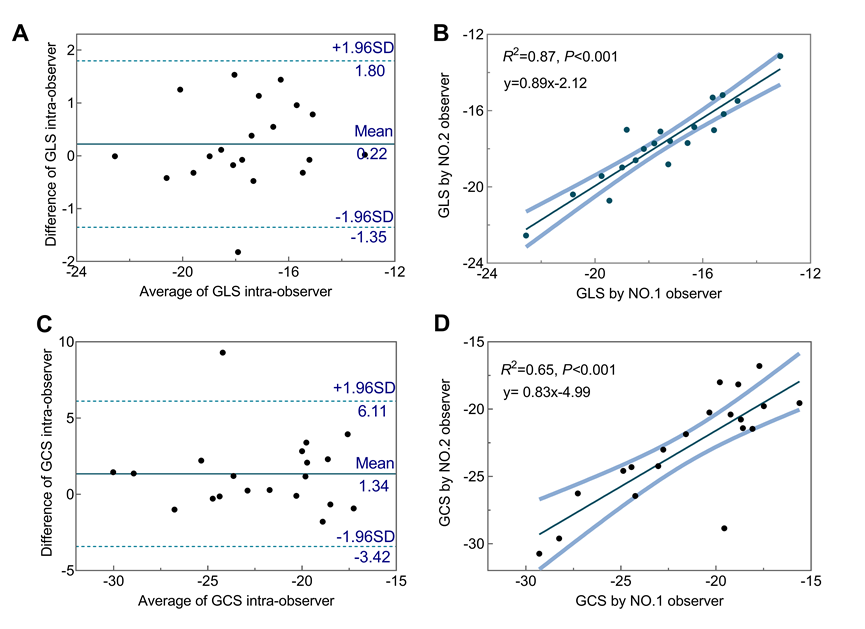

Supplement: Supplementary Figure 1 — The repeatability evaluation adopted a linear correlation analysis and Bland-Altman plots. (A) The mean (±SD) difference was 0.22 (±0.80) for repeated measurements of GLS taken by two independent observers. (B) The linear correlation of GLS between 2 observers showed that R2 = 0.87. (C) The mean (±SD) difference was 1.34 (±2.43) for repeated measurements of GCS taken by two independent observers. (D) The linear correlation of GCS between 2 observers showed that R2 = 0.65. [file Image1.tif]
